# Functional characterization of 16 variants found in the LDL receptor gene

**DOI:** 10.1016/j.jlr.2025.100873

**Published:** 2025-08-12

**Authors:** Kateřina Konečná, Tereza Přerovská, Tomáš Loja, Lenka Fajkusová, Jana Koutná, Michal Kramárek, Ana Catarina Alves, Mafalda Bourbon, Tomáš Freiberger, Lukáš Tichý

**Affiliations:** 1 Centre of Molecular Biology and Genetics, University Hospital Brno, Brno, Czech Republic; 2 National Centre for Biomolecular Research, Faculty of Science, Masaryk University, Brno, Czech Republic; 3 Faculty of Medicine, Masaryk University, Brno, Czech Republic; 4 Central European Institute of Technology, Masaryk University, Brno, Czech Republic; 5 Centre for Cardiovascular Surgery and Transplantation, Brno, Czech Republic; 6 Departamento de Promoção da Saúde e Doenças não transmissíveis, Instituto Nacional de Saúde Dr. Ricardo Jorge, Lisboa, Portugal; 7 BioISI – Biosystems & Integrative Sciences Institute, Faculty of Sciences, University of Lisboa, Lisboa, Portugal

**Keywords:** dyslipidemias, lipoproteins/receptors, lipoproteins, LDL, LDL/metabolism, familial hypercholesterolemia, functional characterization, flow cytometry

## Abstract

Familial hypercholesterolemia (FH) is a disorder of cholesterol metabolism characterized by elevated LDL-cholesterol levels. The most common cause of FH is pathogenic variants in the LDL receptor (*LDLR*) gene. To shed light on the functional impact of selected *LDLR* variants, we functionally characterized 16 *LDLR* genetic variants alongside 10 control variants. We performed in vitro assays based on transient expression of WT and mutant LDLRs in LDLR-deficient Chinese hamster ovary cells. We used flow cytometry to analyze the relative amount of LDLRs expressed on the cell surface and the relative amount of internalized LDL. In addition, we analyzed the expression and maturation of LDLR protein by Western blotting. Of the 16 studied variants, two variants (p.(Asn272Thr) and p.(Arg574Leu)) did not exhibit a defect in LDLR function, one variant (p.(Ala540Thr)) exhibited a defect in LDL binding and/or internalization despite normal LDLR cell surface expression, and the remaining 13 variants had a detrimental effect on both LDLR cell surface expression and LDL internalization. The information presented in this study contributes to the clinical classification of *LDLR* variants and a more precise diagnosis of FH patients, highlighting the type of defect each variant produces.

One of the main genetic factors contributing to CVD are genetic variants in genes associated with familial hypercholesterolemia (FH [Mendelian Inheritance in Man: 143890]) (1). FH is a genetic disorder of cholesterol metabolism that causes elevated levels of LDL-cholesterol in the blood since birth. Plasma LDL-cholesterol levels are directly correlated to the risk of atherosclerotic CVD (2). The elevated number of years of exposure to high LDL-cholesterol concentrations in FH patients leads to a greater cardiovascular risk compared with people without FH (3).

The most common cause of FH is variants in the LDL receptor (*LDLR*) gene (Hugo Gene Nomenclature Committee: 6547; Mendelian Inheritance in Man: 606945) (4). The LDLR binds LDL-cholesterol and facilitates its internalization through receptor-mediated endocytosis. The *LDLR* gene encodes a preprotein that is 860 amino acids long, including a 21 amino acid long signal sequence that is later cleaved off, giving rise to a mature protein of 839 amino acids (5).

The LDLR protein consists of five domains and a signal sequence. The signal sequence is situated at the N-terminal end of the protein. The first domain of the mature protein is the ligand-binding domain, which comprises seven repeats (R1-R7) (UniProt Knowledgebase, accession number: P01130, accessed: April 22, 2024, https://www.uniprot.org/uniprotkb/P01130/). The second LDLR domain is the epidermal growth factor (EGF) precursor homology domain, which was named after its homology to the mouse EGF precursor (6). The next domain is the O-linked sugar domain, which undergoes O-linked glycosylation in the Golgi apparatus (7). This domain is followed by the transmembrane domain and the cytosolic domain. The cytosolic domain contains the NPVY motif, which is necessary for receptor internalization (8, 9, 10).

LDLR life cycle includes synthesis in the endoplasmic reticulum (ER), transport to the cell surface, and many cycles of internalization and return to the cell surface. The first step in LDLR biosynthesis is the synthesis of the signal sequence in the cytoplasm. The signal sequence is then recognized by the signal recognition particle, which targets the protein to the ER for synthesis (11). LDLR undergoes folding in the environment of the ER. Correctly folded LDLR proteins continue to the Golgi apparatus. This transition is marked by an increase in LDLR molecular weight (MW) due to the addition of O-linked sugars in the Golgi (7). The precursor form of LDLR present in the ER has an apparent MW of approximately 120 kDa, whereas the mature form exhibits an MW of approximately 160 kDa (7, 12). After reaching the cell membrane, LDLR clusters in clathrin-coated pits, ready to bind LDL. Clathrin-coated pits are continuously internalized, regardless of LDL binding (13, 14, 15). After being internalized, LDLR enters the endosome, where it undergoes a conformational change due to lower pH and releases the bound LDL particle. While the LDL particle continues to the lysosome for degradation, LDLR is recycled back to the cell surface (16).

*LDLR* variants have been divided into five main classes based on the stage of the LDLR life cycle that is affected by the variant. Class 1 variants produce no detectable LDLR protein (null variants). Class 2 variants cause the LDLR protein to be retained in the ER. They have been divided into class 2a, which is marked by complete retention in the ER, and class 2b, which causes partial retention in the ER associated with a decreased amount of LDLR on the cell surface. Class 3 variants encode proteins defective in LDL binding. Class 4 variants inhibit the internalization of LDLR. Class 5 variants affect LDLR recycling. They prevent LDLR from returning to the cell surface after being internalized, causing LDLR to be degraded in the lysosome (17, 18). Later, two other classes of *LDLR* variants were proposed: variants affecting polarized targeting of the LDLR protein (19), and variants in the transmembrane domain preventing normal membrane insertion of LDLR (11, 20, 21).

Over 4,500 variants in the *LDLR* gene have been reported in the ClinVar database as of July 2025, including more than 1,700 missense variants (https://www.ncbi.nlm.nih.gov/clinvar/?term=ldlr%5Bgene%5D) (22). However, about half of *LDLR* missense variants are classified as variants of unknown significance (VUS) even after expert review by the FH Variant Curation Expert Panel (VCEP) (ClinGen—Clinical Genome Resource—*LDLR* gene, accessed July 8, 2025, https://search.clinicalgenome.org/kb/genes/HGNC:6547). The goal of this study was to provide functional evidence for the pathogenicity assessment of selected sequence variants in the *LDLR* gene, which may allow them to be reclassified as pathogenic or benign.

## Materials and Methods

### Selection of variants

Variants for functional characterization were chosen from known *LDLR* variants found in Czech FH patients (23, 24), which were missing a level 1 functional study as defined by the ClinGen FH VCEP specifications to the American College of Medical Genetics and Genomics (ACMG)/Association for Molecular Pathology (AMP) Variant Classification Guidelines (25). Apart from these 16 variants, 10 previously characterized variants belonging to different classes of *LDLR* variants, as well as WT *LDLR*, were used as controls. Variants p.(Ala391Thr) and p.(Thr726Ile) were chosen as benign controls based on their benign classification in ClinVar (variation accession VCV000183138.45 and VCV000036461.54, respectively), which has been reviewed by the FH VCEP (canonical allele identifiers CA023426 and CA023649, respectively). Pathogenic control variants were chosen based on previously published functional studies delineating their variant class. Control variants belonged to one class except for p.(Val429Met). Variant p.(Val429Met) was used as a control for the combination of classes 2b and 5 based on two previous publications: Ranheim *et al.* (26) classified this variant as class 5 based on its colocalization with endosomes, whereas Dušková *et al.* (27) showed that the variant was associated with increased expression of ER-resident chaperones, which is a marker for a folding defect typical for class 2 variants. The list of *LDLR* variants used in this study can be found in Table 1.

**Table 1:** Variants functionally characterized in the current study

Protein change^a^	DNA change^b^	Variant class	Previous functional study
Control variants			
p.(Ala391Thr)	c.1171G>A	Benign control	(28)
p.(Thr726Ile)	c.2177C>T	Benign control	(29)
p.(Ser691Ter)	c.2072C>A	Class 1	None
p.(Ile441Thr)	c.1322T>C	Class 2a	(30)
p.(Gly565Val)	c.1694G>T	Class 2a	(17, 26, 27, 31)
p.(Arg406Trp)	c.1216C>T	Class 2b	(30)
p.(Cys155Tyr)	c.464G>A	Class 3	(32)
p.(Asn825Lys)	c.2475C>A	Class 4	(32)
p.(Arg416Trp)	c.1246C>T	Class 5	(27, 30, 32)
p.(Val429Met)	c.1285G>A	Class 2b+5	(17, 26, 27)
Variants under study			
p.(Leu16Pro)	c.47T>C	—	None
p.(Asp90Tyr)	c.268G>T	—	Compound heterozygous patient’s fibroblasts, ^125^I-LDL assays (33)
p.(Glu140Asp)	c.420G>C	—	None
p.(Cys143Trp)	c.429C>G	—	None
p.(Gly149Cys)	c.445G>T	—	None
p.(Gly219del)	c.654_656del	—	Homozygous patient’s fibroblasts, ^125^I-LDL assays (denoted as ΔG197) (17)
p.(Asn272Thr)	c.815A>C	—	None
p.(Cys364Ser)	c.1091G>C	—	None
p.(Gln384_Asp386del)	c.1151_1159del	—	None
p.(Phe403del)	c.1207_1209del	—	None
p.(Ala431Thr)	c.1291G>A	—	Homozygous patient’s fibroblasts, ^125^I-LDL assays (18); heterologous (COS) cells (34)
p.(Val523Met)	c.1567G>A	—	Homozygous patient’s fibroblasts, ^125^I-LDL assays (18); homozygous patient’s fibroblasts, ^125^I-LDL assays (35); heterozygous patient’s lymphocytes (36)
p.(Ala540Thr)	c.1618G>A	—	Heterozygous patient’s fibroblasts, immunoblot, and ^125^I-LDL assays (37)
p.(Arg574Leu)	c.1721G>T	—	None
p.(Pro608Ser)	c.1822C>T	—	None
p.(Cys803Arg)	c.2407T>C	—	None

a Protein level annotation is based on the reference sequence NP_000518.1.

b DNA level variant annotation is based on the reference sequence NC_000019.9 (NM_000527.5).

### Preparation of plasmids

The pcDNA3-LDLR plasmid contained the WT *LDLR* complementary DNA (cDNA) located after a strong constitutive promoter. The moxGFP plasmid, encoding the moxGFP protein, was kindly provided by Dr Erik Snapp and obtained through Addgene (Addgene plasmid #68070). The moxGFP protein is a monomeric variant of the GFP optimized for use in oxidizing environments to eliminate the risk that the dimerization of the fluorescent tag would affect the localization and function of the fusion partner (38). Subsequently, the pcDNA3-LDLR-moxGFP plasmid, which encodes moxGFP fused to the C terminus of LDLR, was prepared by cloning the moxGFP gene from the moxGFP plasmid into the pcDNA3-LDLR plasmid using GeneArt™ Gibson Assembly HiFi Cloning Kit (Invitrogen). The two proteins were connected by a GGGGSGGGGS linker.

Sequence variants were introduced into the *LDLR* cDNA inside the pcDNA3-LDLR and pcDNA3-LDLR-moxGFP plasmids using the QuikChange Lightning mutagenesis kit (Agilent) according to the manufacturer’s instructions. Primers used for mutagenesis can be found in supplemental Table S1. Following mutagenesis, the sequence of the whole *LDLR* cDNA, including the cytomegalovirus promoter, was verified by Sanger sequencing. Transfection-grade plasmids were isolated with the ZymoPURE Plasmid Miniprep Kit (Zymo Research).

### Cell cultivation and transfection

An LDLR-deficient cell line prepared by mutagenesis of the Chinese hamster ovary (CHO) cell line (known as the CHO-ldlA7 cell line) was kindly provided by Dr Monty Krieger, who had created the cell line in 1983 (39) (the CHO-ldlA7 cell line is denoted as “320-7a-1” in this publication). CHO-ldlA7 cells were cultured in Ham's F-12 Nutrient Mix with GlutaMAX Supplement (Gibco), supplemented with 10% FBS (“complete medium”), and passaged using the Gibco TrypLE Express Enzyme. The cells were cultured in an incubator at 37°C and 5% CO_2_. Transfection was performed in Ham's F-12 Nutrient Mix with GlutaMAX Supplement without the addition of FBS (“serum-free medium”). Transfection complexes were prepared in the Opti-MEM I Reduced Serum Medium (Gibco).

Transfection was performed using a transfection grade linear polyethyleneimine, MW 25,000 (PEI 25K™) manufactured by Polysciences. To prepare the transfection reagent, powdered PEI was dissolved in Milli-Q water at a final concentration of 1 mg/ml. The pH was adjusted to < 2 with HCl, and the solution was stirred for 2–3 h until it became clear. Then the pH was adjusted to 6.9 by adding NaOH, and the solution was sterile filtered through a 0.22 μm membrane. The sterilized PEI solution was aliquoted and frozen at −20°C. After thawing, a PEI aliquot was kept in a fridge and discarded after 2–4 weeks.

For flow cytometry analyses, the transfection was performed in a 24-well plate, whereas for Western blotting, the transfection was performed in a 6-well plate.

#### Transfection with PEI in a 24-well plate

One day before transfection, CHO-ldlA7 cells were seeded in a 24-well plate so that they would be approximately 50–70% confluent at the time of transfection. One hour before the addition of transfection complexes, the plate was washed once with PBS, and the medium was exchanged for 1 ml of the serum-free medium. Cells were returned to the incubator for 1 h to become accustomed to the new medium. Transfection complexes were prepared by mixing 1.5 μg plasmid DNA and 3 μl of the PEI solution (stock concentration: 1 mg/ml) per well. First, plasmid DNA and PEI were separately diluted in 50 μl of room temperature Opti-MEM per well, vortexed, and incubated for 5 min at room temperature. Then, 50 μl of diluted PEI was added dropwise to 50 μl of diluted DNA (per well). The transfection complexes were immediately vortexed for 5–10 s at medium speed. After mixing, each premix was incubated at room temperature for 12 min. At the end of incubation, 95 μl of transfection complexes were added dropwise into each well. The plate was gently rocked and then returned to the incubator. One day after transfection, the medium was exchanged for the complete medium, and the cells were cultured for one more day. Transfection efficiency was approximately 10–25% in different experiments. Flow cytometry analyses were performed 2 days after transfection.

#### Transfection with PEI in a 6-well plate

One day before transfection, CHO-ldlA7 cells were seeded in a 6-well plate so that they would be approximately 20–50% confluent at the time of transfection. One hour before the addition of transfection complexes, the plate was washed once with PBS, and the medium was exchanged for 2 ml of the serum-free medium. Transfection complexes were prepared by mixing 3 μg plasmid DNA and 6 μl of the PEI solution (stock concentration: 1 mg/ml) per well. First, plasmid DNA and PEI were separately diluted in 100 μl of room temperature Opti-MEM per well, vortexed, and incubated for 5 min at room temperature. Then, 100 μl of diluted PEI was added dropwise to 100 μl of diluted DNA (per one well). The transfection complexes were immediately vortexed for 5–10 s at medium speed. After mixing, each premix was incubated at room temperature for 12 min. At the end of incubation, 200 μl of transfection complexes were added dropwise into each well. The plate was gently rocked and then returned to the incubator. One day after transfection, the medium was exchanged for the complete medium, and the cells were cultured for one more day before the preparation of lysates.

### Flow cytometry analysis of LDLR cell surface expression

Cells were transfected in a 24-well plate as described above. Each well was transfected with the pcDNA3-LDLR-moxGFP plasmid encoding one *LDLR* variant. To improve accuracy in each experiment, each variant was transfected in triplicate wells, and the results from all three wells were averaged. The experiment was performed at least three times for each variant (3 × 3 wells per variant). Two days after transfection, the cells were detached using Accutase (Invitrogen), transferred into 1.5 ml tubes, and washed twice with 1 ml of fluorescence-activated cell sorting (FACS) buffer consisting of PBS with 2% FBS and 10 mM EDTA. The cells were stained with anti-LDLR antibody C7 conjugated with allophycocyanin (APC) manufactured by Novus Biologicals (“anti-LDLR-APC antibody”; catalog no.: NBP1-78159APC) as follows. The cell pellet was resuspended in 100 μl of the staining solution consisting of FACS buffer and the anti-LDLR-APC antibody at a concentration of 0.008 μg/μl. The cells were stained for 1.5 h at 5°C in the dark. After staining, cells were washed twice with 1 ml of FACS buffer and resuspended in SYTOX Blue dead cell staining solution consisting of SYTOX Blue (Invitrogen) in FACS buffer (1:500 dilution).

The samples were analyzed on the BD FACSVerse flow cytometer using BD FACSuite software, v1.0.6 (BD Biosciences). About 40,000 events were measured for each sample. A population of single cells was gated in a plot of forward scatter height versus forward scatter area. Living cells were gated based on SYTOX Blue intensity. A sample containing LDLR+GFP- cells (obtained by transfecting CHO-ldlA7 cells with the WT pcDNA3-LDLR plasmid without moxGFP and labeling them with the anti-LDLR-APC antibody) was used as a fluorescence-minus-one (FMO) control to set the gate for GFP positivity. To separate transfected from nontransfected cells, only GFP+ cells were used in subsequent data analysis. From the population of transfected (GFP+) cells in each sample, the median fluorescence intensity (MFI) of APC was obtained. In each experiment, results from three replicate wells transfected with the same variant were averaged, and the average values were converted to percentages by relating them to the result of the control benign variant p.(Ala391Thr) obtained in the same experimental run. Because the absolute MFI obtained on different days can vary due to various factors, we did not compare absolute MFI values obtained on different days but instead compared percentages related to p.(Ala391Thr). After repeating the experiment at least three times for each variant, the percentages obtained for the same variant were averaged. The benign variant p.(Ala391Thr) was chosen as a reference to be used in every experiment instead of WT because the handling of this variant more closely corresponded to the handling of other variants, whereas the WT plasmid was missing the mutagenesis step. WT was in turn used as a reference for statistical testing because the results for variant p.(Ala391Thr) were normalized to 100% in every experiment and thus did not show any variability.

In every experiment, a control sample containing cells transfected with the WT pcDNA3-LDLR-moxGFP plasmid without the addition of the anti-LDLR-APC antibody was used. With the addition of SYTOX Blue, this sample served as an FMO control without APC. In addition, every flow cytometry experiment included a sample transfected with the pcDNA3-LDLR plasmid without the moxGFP fusion with added anti-LDLR-APC antibody and SYTOX Blue to serve as an FMO control without moxGFP. In some experiments, LDLR-deficient cells with added anti-LDLR-APC antibody were used to assess the level of nonspecific antibody binding. LDLR-deficient cells consisted of either mock-transfected cells (treated with the transfection reagent without the addition of plasmid DNA) or cells transfected with the moxGFP plasmid (encoding the moxGFP fluorescent protein without LDLR). According to LDLR-deficient controls, nonspecific antibody binding was low (less than 1% of cells were observed within the LDLR+ gate).

### Flow cytometry analysis of LDL internalization

Cells were transfected with the pcDNA3-LDLR-moxGFP plasmid encoding different *LDLR* variants in a 24-well plate as described above. In every experiment, each variant was transfected in duplicate wells, and the results from both wells were averaged. Each experiment was performed at least three times for each variant (3 × 2 wells per variant). The internalization assay was performed 2 days after transfection. To avoid the presence of unlabeled LDL originating from FBS, the complete medium was exchanged for Ham's F-12 Nutrient Mix with GlutaMAX, supplemented with 0.3% BSA instead of FBS. LDL particles labeled with pHrodo Red (pHrodo Red-LDL supplied by Invitrogen, 1 mg/ml, catalog no.: L34356) were diluted in the medium at the volume of 5 μl of pHrodo Red-LDL and 500 μl of BSA-containing medium per well. The medium was aspirated from the wells and exchanged for 500 μl of pHrodo Red-LDL-containing medium. The plate was incubated in a CO_2_ incubator at 37°C for 20–30 min. Then the plate was washed with PBS, and the cells were detached using Accutase and transferred into 1.5 ml tubes. The cells were washed two times with FACS buffer and resuspended in SYTOX Blue dead cell staining solution. The samples were analyzed on the BD FACSVerse flow cytometer and gated as described above. Transfected (GFP+) cells were gated, and the MFI of pHrodo Red in duplicate samples transfected with the same variant was averaged. The average values were converted to percentages by relating them to the result of the benign variant p.(Ala391Thr), as described for the flow cytometry analysis of LDLR cell surface expression.

In every internalization experiment, a control sample containing cells transfected with the WT pcDNA3-LDLR-moxGFP plasmid without the addition of pHrodo Red-LDL was used. With the addition of SYTOX Blue, this sample served as an FMO control without pHrodo Red. In addition, every flow cytometry experiment included a sample transfected with the pcDNA3-LDLR plasmid without the moxGFP fusion, followed by the addition of pHrodo Red-LDL and SYTOX Blue dead cell staining solution, to serve as an FMO control without moxGFP. Single-stained controls were used to set spillover compensation. As a control for LDLR-independent uptake of pHrodo Red-LDL, nontransfected or mock-transfected cells were incubated with pHrodo Red-LDL.

### Western blotting

Cells were seeded in 6-well plates and transfected with the pcDNA3-LDLR plasmid encoding different *LDLR* variants (without moxGFP fusion) as described above. Two days after transfection, the extraction of protein lysates was performed. The plate was washed two times with cold PBS while the plate was kept on ice. The PBS was exchanged for 150 μl of ice-cold lysis buffer consisting of RIPA Lysis and Extraction Buffer (Thermo Fisher Scientific) with the addition of Halt™ Protease Inhibitor Cocktail and Halt™ Phosphatase Inhibitor Cocktail (Thermo Fisher Scientific). Cells were detached from the surface by repeated pipetting up and down with the lysis buffer, transferred to an ice-cold 1.5 ml tube, and incubated for 30 min on ice. Then the samples were centrifuged at 14,000 *g*, 4°C for 30 min. The supernatant was transferred to a new tube, and the lysates were flash-frozen in liquid nitrogen and kept at −80°C.

Protein content was measured by a Pierce™ BCA Protein Assay Kit (Thermo Fisher Scientific) according to the product manual. Briefly, for measurement of the calibration line, BSA in concentrations of 0, 0.025, 0.125, 0.25, 0.5, 0.75, 1, 1.5, and 2 mg/ml was used. About 50 μl of standards or samples were mixed with 1 ml of the BCA reagent. In the blank measurement, only the lysis buffer was used. The solutions were incubated at 37°C for 30 min, and the absorbance was read at 562 nm.

For the PAGE separation, samples were mixed with 4X Laemmli sample buffer with a reducing agent, boiled for 10 min, and 20 μl were loaded on 7.5% polyacrylamide gels. Gels were run at a constant current of 100 V for 15 min, and then the current was increased to 150 V for approximately 45 min.

Separated proteins were transferred to nitrocellulose membranes, which were blocked with 5% low-fat milk in TBS with 0.05% Tween-20 (v/v; TBST) overnight at 4°C on a rocking platform. The membranes were incubated with either an anti-LDLR monoclonal antibody produced in mouse (1:1,000 dilution; catalog no.: MA5-38556; Invitrogen) or an anti-GAPDH antibody produced in mouse (1:1,000 dilution; catalog no.: MA5-15738; Invitrogen) or in rabbit (1:5,000 dilution; catalog no.: G9545; Sigma-Aldrich) in 5% low-fat milk in TBST for 1.5 h at room temperature on a rocking platform at 100 rpm. After washing with TBST three times for 15 min at room temperature on a rocking platform, blots were incubated with either goat anti-mouse IgG (H + L) secondary antibody (1:5,000 dilution; catalog no.: g-21040; Invitrogen) or goat anti-rabbit IgG (whole molecule) secondary antibody (1:10,000 dilution; catalog no.: A0545; Sigma-Aldrich) coupled to horseradish peroxidase in 5% low-fat milk in TBST for 1.5 h at room temperature on a rocking platform at 100 rpm. After washing as described above, the membranes were developed in SuperSignal West Femto Maximum Sensitivity Substrate (Thermo Fisher Scientific) for 5 min at room temperature, and chemiluminescence was detected by the Fusion FX imaging system (Vilber Lourmat).

The intensity of individual bands was quantified using GelAnalyzer, v19.1. First, lanes and bands were manually selected. Then, the background was automatically detected (mode: morphological). The value of the “raw volume” was collected for each band. To calculate the ratio of the mature to the precursor form of LDLR, the “raw volume” of the 160 kDa band (corresponding to the mature form of LDLR) was related to that of the 120 kDa band (precursor form).

LDLR protein expression was assessed semiquantitatively. Small differences in protein expression could not be quantified because variations in transfection efficiency between different experiments prevented reliable normalization of LDLR expression to GAPDH. Thus, only semiquantitative observations of the combined strength of both LDLR bands were reported.

### Statistical analysis

Dixon’s Q test was used to identify outliers (α = 0.05), which led to the removal of one observation (142% LDLR cell surface expression for p.(Ala540Thr)). The results for each variant were compared with WT using ANOVA with Dunnett's post hoc test in GraphPad Prism, version 10.2.3 (GraphPad Software, Inc). When numerical results are reported in the text, they are presented as the mean of three or more experiments ± SD. The values of the mean, SD, and the number of replicates for all variants can be found in supplemental Tables S2, S3 and S4.

## Results

### Flow cytometry analysis of LDLR cell surface expression

Flow cytometry was used to quantify the amount of LDLR on the cell surface, which is decreased in variant classes 1, 2, and 5. LDLR cell surface expression was quantified using a primary anti-LDLR antibody conjugated to APC, which binds only LDLRs present on the cell surface when used on nonpermeabilized living cells. As controls, WT *LDLR* and 10 previously characterized variants belonging to each of the *LDLR* variant classes were also analyzed.

**Fig. 1:**
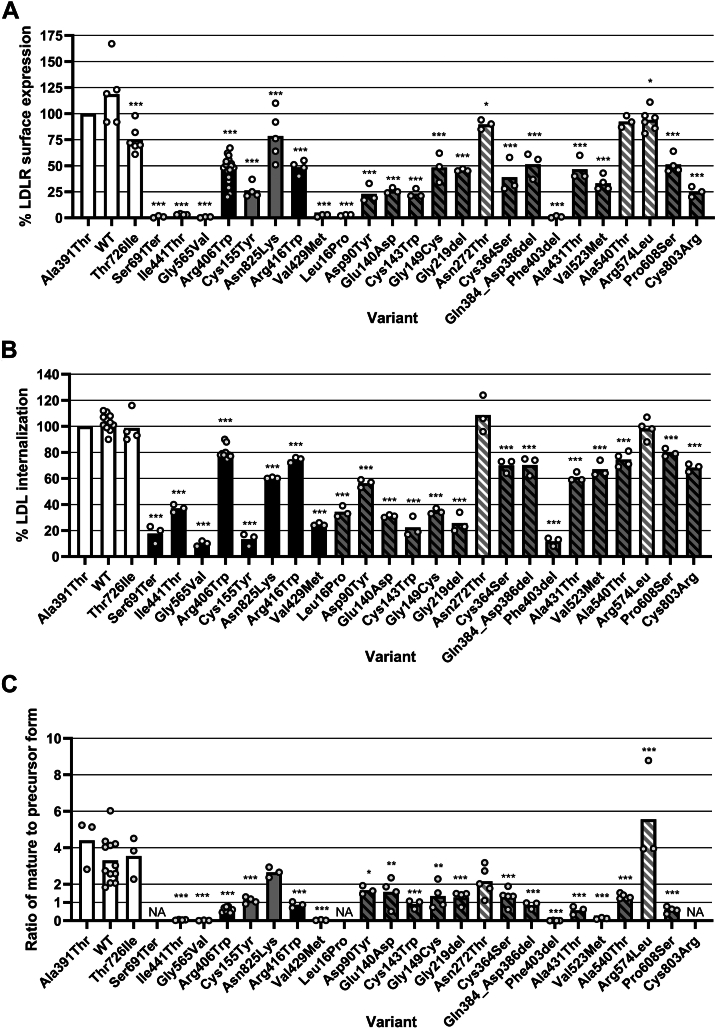
Results of the flow cytometry analysis of LDLR cell surface expression (A), LDL internalization (B), and the Western blot analysis of LDLR maturation (C). The bars show the mean result of all experiments, whereas the dots show the results of individual experiments. Each variant was analyzed in at least three independent experiments. The bars are color-coded according to the variant’s functional effect: white—benign controls; black—pathogenic controls defective in the respective assay; gray—pathogenic controls expected to show no defect in the respective assay; gray with black hatching—studied variants that exhibited a functional defect in the respective assay; gray with white hatching—studied variants that did not exhibited a functional defect in the respective assay. All variants were compared with WT using ANOVA with Dunnett’s post hoc test (∗∗∗*P* < 0.001; ∗∗*P* < 0.01; and ∗*P* < 0.05). The mature-to-precursor ratio was not analyzed for a nonsense variant producing a truncated protein that was detected as only one band (p.(Ser691Ter)) or variants whose bands were too weak for quantification (p.(Leu16Pro) and p.(Cys803Arg)). For the flow cytometry analyses, the results of each experiment were converted to percentages by relating them to the benign control p.(Ala391Thr). The value of the mean, standard deviation, and the number of replicates for all three graphs can be found in supplemental Tables S2, S3, and S4. The interpretation of these results is further discussed in the Discussion section and summarized in Figure 3. NA, not analyzed.

The thresholds for a functional defect (<70%) and no defect (>90%) were based on *LDLR*-specific ACMG/AMP guidelines (25). Based on these thresholds, one of the benign controls (p.(Thr726Ile)) and the class 4 control p.(Asn825Lys) fell into the ambiguous “gray zone” between 70% and 90% (Fig. 1A and supplemental Table S2).

Two newly characterized variants exhibited normal cell surface expression (p.(Ala540Thr) and p.(Arg574Leu)), and one variant was just below the threshold for normal function, falling into the gray zone (p.(Asn272Thr with 90 ± 5%).

Four control variants (p.(Ser691Ter), p.(Ile441Thr), p.(Gly565Val), and p.(Val429Met)) and two newly characterized variants (p.(Leu16Pro) and p.(Phe403del)) caused a nearly complete absence of LDLRs on the cell surface (<10%), whereas the rest of the variants caused a slight to moderate decrease in LDLR cell surface expression (10–69%). Unexpectedly, the class 3 control variant, p.(Cys155Tyr), which was previously characterized as having normal cell surface expression combined with lowered LDL binding (32), exhibited markedly decreased LDLR cell surface expression in our experimental setup.

### Flow cytometry analysis of LDL internalization

Flow cytometry was also used to assess the ability of mutant LDLR proteins to internalize pHrodo Red-labeled LDL particles. The use of the pH-sensitive dye pHrodo Red allowed for the selective detection of internalized LDL without detecting LDL bound to LDLR on the cell surface, proving that the mutated LDLR is able to both bind and internalize LDL.

Pathogenic controls belonging to classes 1, 2, and 5 exhibited a higher level of internalization than expected (Fig. 1B and supplemental Table S3). Due to this, the thresholds for a functional defect and normal function were shifted to <80% and >95%, respectively. Control variants class 2a, which have nearly no LDLR on the cell surface, exhibited LDL internalization of 10–37%, hence the threshold for a severe defect of internalization was set to <40%.

Of the newly characterized variants, two variants (p.(Asn272Thr) and p.(Arg574Leu)) internalized LDL at a level similar to WT, whereas the remaining variants showed reduced LDL internalization (Fig. 1B and supplemental Table S3). A severe reduction in LDL internalization was caused by variants p.(Leu16Pro), p.(Glu140Asp), p.(Cys143Trp), p.(Gly149Cys), p.(Gly219del), and p.(Phe403del).

### Western blotting

To complement the flow cytometry analysis of LDLR cell surface expression, we also analyzed LDLR protein expression and LDLR maturation by Western blotting. A decreased cell surface expression is expected to correlate either with reduced protein expression or a decreased ratio of the mature to the precursor form of LDLR. Although the fluorescent tag used for flow cytometry assays (moxGFP) has been designed to mitigate the risk of experimental artifacts, we decided to verify LDLR maturation by Western blotting without the fluorescent tag.

Western blotting allows for the determination of the mature-to-precursor ratio by comparing the 160 kDa band of mature LDLR to the 120 kDa band of its precursor form. Western blotting could not distinguish between a maturation defect (class 2b) and a recycling defect (class 5) because both the class 2b control (p.(Arg406Trp)) and the class 5 control (p.(Arg416Trp)) had very similar results. Since benign controls (WT, p.(Ala391Thr), and p.(Thr726Ile)) exhibited a mature-to-precursor ratio of > 2.0 and defective controls (p.(Arg406Trp) and p.(Arg416Trp)) showed a ratio of < 1.0, we chose the mature-to-precursor ratio of >2.0 as a threshold for normal LDLR maturation and recycling, whereas a ratio <1.0 was considered a sign of a moderate defect of LDLR maturation or recycling. In addition, based on the results of class 2a control variants, a ratio of < 0.2 was considered to mark a severe defect. Many of the studied variants exhibited a mean ratio between 1.0 and 2.0, which was challenging to interpret because none of the controls, except p.(Cys155Tyr), showed a ratio in this range. However, in the newly characterized variants, a mature-to-precursor ratio between 1.0 and 2.0 seemed to correlate with decreased LDLR cell surface expression as determined by flow cytometry.

**Fig. 2:**
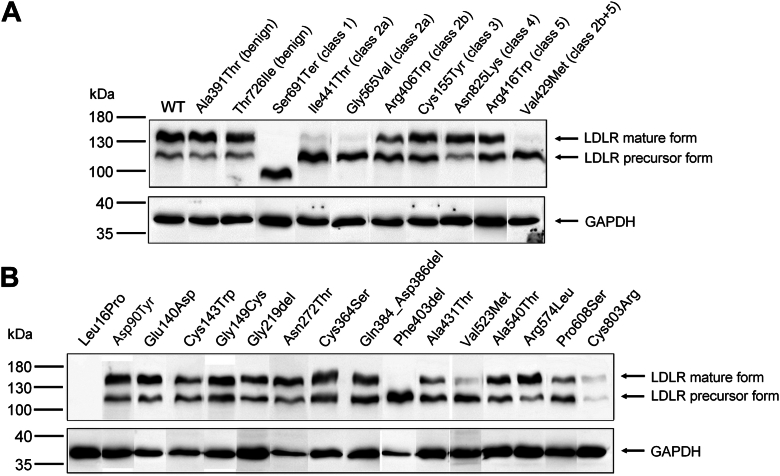
Western blot analysis of *LDLR* variants. The figure is separated into controls (A) and variants under study (B). The figure was constructed by rearranging single lanes from images of nine separate gels. Each variant was analyzed in at least three biological replicates. One representative result for each variant is shown. Anti-GAPDH antibody was used as a loading control.

Two variants exhibited decreased protein expression: the p.(Leu16Pro) variant was nearly undetectable by Western blotting, whereas the p.(Cys803Arg) variant could only be detected as two faint bands. All the other variants produced strong bands (Fig. 2).

Normal maturation was observed for benign controls, the class 4 control p.(Asn825Lys), and two newly characterized variants (p.(Asn272Thr) and p.(Arg574Leu)) (Figs. 1C and 2 and supplemental Table S4). In accordance with flow cytometry results, variants p.(Ile441Thr), p.(Gly565Val), p.(Val429Met), and p.(Phe403del) appeared on the gel solely in the precursor form. The nonsense variant p.(Ser691Ter) was expressed as one band of decreased MW. Variants that exhibited reduced LDLR cell surface expression (<70%) based on the flow cytometry assay also showed a mature-to-precursor ratio below 2.0, and vice versa, with one exception; p.(Ala540Thr), surprisingly exhibited a mature-to-precursor ratio of 1.33 ± 0.14, suggesting the possibility of a slight defect in LDLR maturation, even though flow cytometry showed normal cell surface expression.

### Summary of results

Of the 16 newly characterized variants, two variants (p.(Asn272Thr) and p.(Arg574Leu)), did not exhibit a defect in LDLR protein expression, maturation, cell surface expression, or LDL internalization; one variant (p.(Ala540Thr)) exhibited decreased internalization combined with normal cell surface expression, whereas the remaining 13 variants caused both a decrease in LDLR cell surface expression and LDL internalization. For variants p.(Leu16Pro) and p.(Cys803Arg), the reduction of LDLR cell surface expression was caused by a reduction in the total amount of the LDLR protein as determined by Western blotting.

**Fig. 3:**
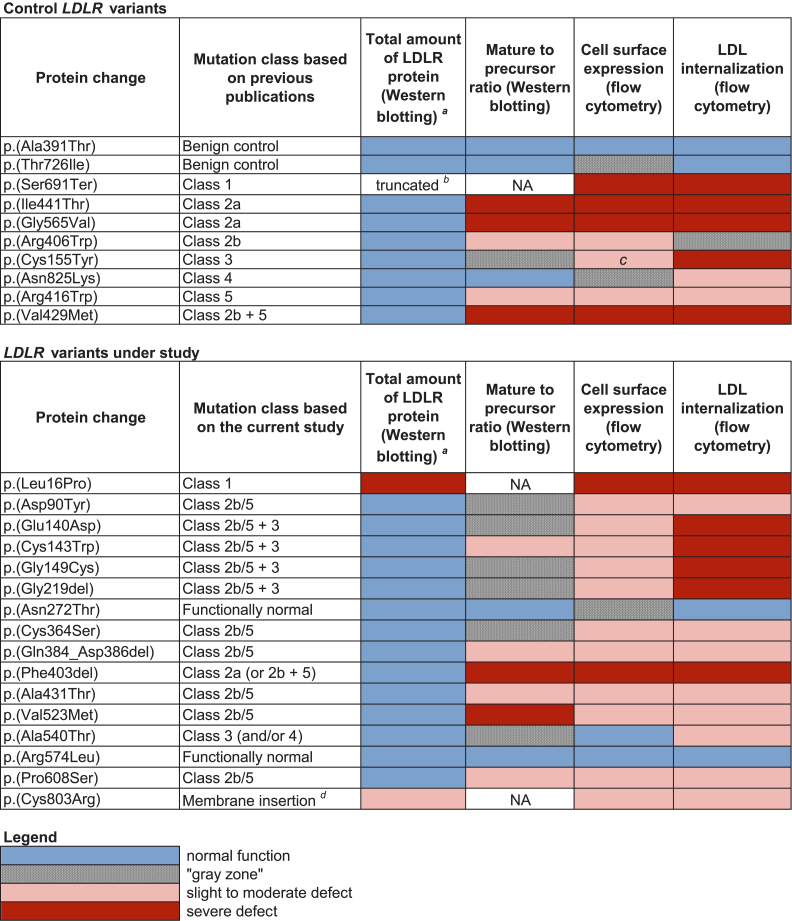
Summary of the results of the current functional study. The results of each assay were classified into four levels of function: normal function (blue), “gray zone,” slight to moderate defect (pink), and severe defect (red). The “gray zone” (gray with hatching) was used for ambiguous results between normal and defective function. The following thresholds were used for each method: Mature-to-precursor ratio (Western blotting): > 2.0—normal; 0.2–2.0—slight to moderate defect; and < 0.2—severe defect. LDLR cell surface expression (flow cytometry): > 90%—normal; 10–69%—slight to moderate defect; and < 10%— severe defect. LDL internalization (flow cytometry): > 95%—normal; 40–79%—slight to moderate defect; and < 40%—severe defect. A reduction in LDL internalization more severe than the reduction in LDLR cell surface expression signifies a defect in binding and/or internalization. The mature-to-precursor ratio was not analyzed for variants where the 120 kDa and 160 kDa LDLR bands were too weak for accurate quantification. ^*a*^This column evaluates the total amount of LDLR protein detected by Western blotting, regardless of its form (mature or precursor). Protein expression was assessed semiquantitatively based on the observed strength of LDLR bands. ^*b*^The nonsense variant p.(Ser691Ter) is expected to cause NMD and abolish LDLR protein expression in vivo; however, the lack of introns in the plasmid construct used in our study prevented NMD and led to the expression of a truncated form of the LDLR protein. ^*c*^The class 3 control p.(Cys155Tyr) was expected to exhibit normal cell surface expression based on a previous publication; however, our results showed decreased cell surface expression (for details, see Discussion). ^*d*^Variant p.(Cys803Arg) belongs to the unnumbered class of variants interfering with the membrane insertion of LDLR (11, 20, 21). NA, not analyzed.

Based on a combination of results of different functional assays, the studied variants were divided into different classes of *LDLR* variants (Fig. 3). A severe defect of LDLR cell surface expression is associated with variant classes 1 and 2a, whereas a slight to moderate defect is associated with classes 2b and 5. Class 1 can be differentiated from class 2a based on the lack of LDLR protein expression as determined by Western blotting. However, the methods used in this study could not distinguish between control variants class 2b and class 5. Therefore, all variants with a slight to moderate defect of LDLR cell surface expression are referred to as “class 2b/5.” A reduction of LDL internalization beyond what can be explained by a reduction of LDLR cell surface expression signifies defective LDL binding or internalization (class 3 or 4, respectively). The methods used in the current study could not unambiguously distinguish between a defect of binding and internalization; however, several variants were classified as class 3 due to their location within the LDLR protein (for details, see Discussion).

## Discussion

Sixteen *LDLR* variants were functionally characterized using flow cytometry and Western blotting. The results are summarized in Figure 3.

### Correspondence between the methods

As shown in Figure 1, the numerical results of the LDLR cell surface expression assay and the LDL internalization assay did not perfectly correspond. Many variants exhibited a higher percentage of internalization than the percentage of LDLR surface expression. It is important to note that the percentages express the median fluorescence signal intensity relative to the benign control p.(Ala391Thr), not a percentage of internalized LDL. Thus, the differences between benign and defective controls reflect the assay’s power to detect a functional defect. The discrepancy between these two methods likely means that, despite extensive optimization, the internalization assay had lower power to detect differences between benign and defective variants than the cell surface expression assay. As seen in Figure 3, variants were assigned one of four functional consequences for each assay (normal function, “gray area,” slight to moderate defect, or severe defect). Because the exact percentages did not correlate between the two flow cytometry methods, we compared the results of different methods by comparing the severity of the defect instead of directly comparing percentages.

Moreover, the internalization study seemed to overestimate the amount of internalized LDL, which was especially evident in variants with < 5% surface expression, which showed LDL internalization of 10–37%. However, the internalization assay was still able to detect a statistically significant difference between WT and all the control pathogenic variants.

There are a few possible explanations for the unexpectedly high LDL internalization. First, it could be attributed to LDLR-independent LDL uptake, which has been shown to occur in HepG2 and HuH7 cells (40, 41). Alternatively, cells transfected with pathogenic variants could be utilizing a mechanism to increase LDL uptake in case of a cholesterol deficiency. This could be a combination of LDLR-dependent and LDLR-independent mechanisms. However, in our study, this mechanism cannot be dependent on the increased expression of LDLR because the *LDLR* gene was located behind a constitutive cytomegalovirus promoter whose expression is independent of cholesterol levels. An LDLR-dependent mechanism of increased LDL uptake might hypothetically be mediated by an upregulation of proteins involved in LDLR internalization, such as LDLR adaptor protein 1 (LDLRAP1) or Dab2 (8, 10). The third possible explanation is related to LDLR overexpression on the cell surface. Because LDL is larger than the LDLR (42), there may not be enough space on the cell surface for every LDLR to bind LDL when the cell surface is saturated with receptors. Thus, the relationship between the amount of LDLRs on the cell surface and the amount of internalized LDL might not be directly proportional under the conditions of LDLR overexpression. Alternatively, endogenous proteins mediating LDLR internalization may be depleted in cells overexpressing LDLR on the cell surface. This may allow variants with decreased LDLR cell surface expression to “catch up” to the internalization levels of benign variants.

### Control variant p.(Ser691Ter)

In our Western blot analysis, the control nonsense variant p.(Ser691Ter) was expressed as one band of decreased MW. In vivo, nonsense variants are expected to be degraded by nonsense-mediated decay (NMD). However, NMD is dependent on the presence of exon junction complexes, which form during splicing. Because the plasmid construct contained cDNA without introns, NMD was not triggered, and the protein was expressed as a truncated variant.

The flow cytometry results for the p.(Ser691Ter) variant were inconsistent with expectations. Given the absence of the transmembrane and intracellular domains, the variant was predicted to exhibit no cell surface expression and LDL internalization. While flow cytometry confirmed nearly undetectable LDLR surface expression (1 ± 1%), a small amount of internalized LDL (18 ± 7%) was observed. This internalization could have been attributed to LDLR-independent pathways; however, microscopic examination revealed a small subset of GFP-positive cells in cultures transfected with p.(Ser691Ter), suggesting that some cells may bypass the nonsense variant and express the full-length protein, including the GFP tag fused to the N terminus of LDLR. The bypassing of a nonsense codon could hypothetically be mediated by a tRNA mutation carried by some cells in the culture, potentially facilitated by the genetic instability and heterogeneity of the CHO cell line (43). Alternatively, expression of the full-length protein could be mediated by naturally occurring sporadic stop-codon read-through (44). Despite these observations, Western blotting failed to detect full-length LDLR protein, which may be attributed to the low sensitivity of the method.

### Class 3 control p.(Cys155Tyr)

The results for the class 3 (binding defective) control p.(Cys155Tyr) were unexpected because they differed from the results of a previous study by Etxebarria *et al.* (32), which was the basis for choosing this variant as a class 3 control. The previous publication reported normal cell surface expression combined with defective LDL binding. In contrast, our study observed significantly reduced LDLR cell surface expression (26 ± 8%), suggesting a classification of p.(Cys155Tyr) as both class 2b/5 and class 3.

The discrepancy between the two studies appears to be dependent on gating. Because Etxebarria *et al.* (32) did not mention the inclusion of a fluorescent tag to gate transfected cells, we assumed that they must have gated cells to be analyzed based on the signal from an anti-LDLR antibody. In contrast, our study gated cells based on GFP fluorescence to isolate all transfected cells, including those that did not express LDLR on the cell surface. When gating our data based on LDLR cell surface expression instead of GFP fluorescence, we obtained similar results to those of Etxebarria *et al.* Thus, we concluded that the exclusion of nontransfected cells caused the disagreement between the results of these two studies.

**Fig. 4:**
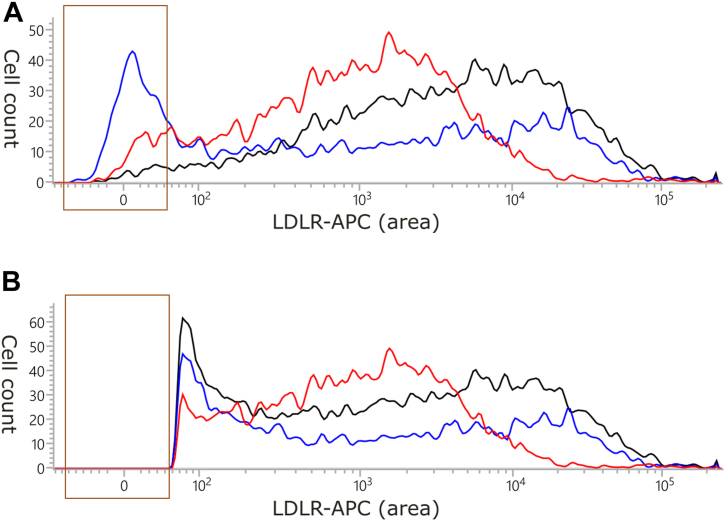
The effects of using the GFP+ gate or the LDLR+ gate to analyze LDLR cell surface expression by flow cytometry. After gating cells based on viability staining, viable cells were gated either based on the fluorescence intensity of moxGFP (GFP+ gate) or the anti-LDLR APC-conjugated antibody (LDLR+) gate. The GFP+ gate was used in our study to analyze transfected cells due to low transfection efficiency, whereas the LDLR+ gate was presumably used in a previous study by Etxebarria *et al.* In graph (A), only cells within the GFP+ gate are plotted, whereas graph (B) shows only cells within the LDLR+ gate. Both graphs show results for the same samples that were all run in the same experiment: black—benign control p.(Ala391Thr), blue—p.(Cys155Tyr), and red—p.(Arg406Trp). The *x*-axis shows the intensity of APC fluorescence, whereas the *y*-axis shows cell count. The brown rectangle highlights the area of the plot most affected by the gating. This figure illustrates how using the LDLR+ gate makes median APC fluorescence similar for variants p.(Cys155Tyr) and p.(Ala391Thr), whereas using the GFP+ gate reveals a large difference between these two variants. In contrast, the distribution of APC fluorescence of the class 2b control variant p.(Arg406Trp) is clearly different from the benign variant with both gates.

A hypothetical explanation is provided below. In our study, LDLR cell surface expression was quantified by calculating the median APC fluorescence of all cells inside the GFP+ gate (assumed to be transfected cells). This included GFP+LDLR- cells that expressed the LDLR-moxGFP fusion protein only intracellularly. The usual proportion of LDLR cells out of GFP+ cells in our experiments was around 10–25% for benign variants and up to 45% for the p.(Cys155Tyr) variant. This means that as much as 45% of cells successfully transfected with the LDLR-moxGFP fusion with the p.(Cys155Tyr) variant had no detectable LDLR protein on the cell surface. However, the remaining cells had a distribution of fluorescence values comparable to that of the benign control (Fig. 4). When gating based on LDLR cell surface expression instead of GFP fluorescence, the surface expression of p.(Cys155Tyr) appeared normal, similar to the benign control, but this overlooked the 45% of transfected cells that did not express LDLR on the cell surface.

The reason why half of the transfected cells expressing p.(Cys155Tyr) cannot support the surface expression of the LDLR protein, whereas the other half can express normal amounts of LDLR on the cell surface, remains unknown. Considering the genetic heterogeneity of the CHO cell line (43), variants in genes encoding chaperones involved in LDLR folding might play a role.

We also considered the option that the p.(Cys155Tyr) variant could have interfered with antibody binding. However, the antibody targets repeat R1 of the binding domain (45), whereas p.(Cys155Tyr) is located in repeat R4, thus the lack of antibody binding cannot be attributed to the p.(Cys155Tyr) variant disrupting the epitope.

Based on our results, p.(Cys155Tyr) can be classified into multiple *LDLR* variant classes. Notably, p.(Cys155Tyr) leads to the loss of a disulfide bond, which is likely to affect folding and cause a decreased LDLR cell surface expression due to retention in the ER (class 2b). Concurrently, the variant affects repeat R4 of the binding domain, which is important not only for LDL binding but also for the release of LDL at low pH, thus potentially affecting LDLR recycling (class 5) (46). The variant was also associated with an increased severity of the binding defect compared with the cell surface expression defect. Based on the results of the current study and the location of this variant, we propose that p.(Cys155Tyr) causes both LDLR retention in the ER (class 2b) and defective LDL binding (class 3), with the possibility of a recycling defect (class 5) as well.

### Functionally normal variants

The c.815A>C (p.(Asn272Thr)) variant is currently classified as a VUS in ClinVar. The current functional study showed no effect on LDLR protein expression, maturation, or the ability to internalize LDL. The value for LDLR cell surface expression (90 ± 5%) was just below the cutoff for no functional defect (>90%), falling into the gray zone between benign and pathogenic functional classification. However, the cell surface expression of the benign control p.(Thr726Ile) was also in the gray zone (Fig. 3).

The c.815A>C variant is located at position -3 of the donor splice site in exon 5, raising the possibility of a splicing effect. However, the functional study could not assess splicing as the plasmid only contained the *LDLR* cDNA. Therefore, the variant’s effect on splicing was assessed using in silico tools. MaxEntScan and SpliceAI predicted no splicing disruption. Thus, we have found no evidence for the pathogenicity of this variant.

The c.1721G>T (p.(Arg574Leu)) variant has been classified as a VUS in ClinVar and ClinGen. Two other variants in the same codon have been reported: c.1721G>A (p.(Arg574His)) and c.1720C>T (p.(Arg574Cys)). All three variants have been predicted to be pathogenic by in silico tools according to ClinGen. Variant p.(Arg574His) has been classified as likely pathogenic by the FH VCEP, though it has not been functionally studied. It was reported to segregate with FH in one family (47). The other variant in the same codon—p.(Arg574Cys)—is currently classified as a VUS. Its functional consequence has been investigated by a level 3 high-throughput assay (48), which indicated no damaging effect of this variant.

According to the results of the current functional study, p.(Arg574Leu) does not affect LDLR protein expression, maturation, cell surface expression, or LDL internalization. However, a study by Junna *et al.* (49) suggested a possible clinical impact, as carriers of p.(Arg574Leu) in a Finnish cohort showed a family history of early onset myocardial infarction and hypercholesterolemia. Even though the information presented by Junna *et al.* is not sufficient for applying any ACMG/AMP criteria, the discrepancy indicates that further verification of the functional effect of this variant is needed. There is a possibility that the variant could belong to a class not investigated by this study. For example, the current functional study was not able to determine the variant’s effect on polarized targeting (19).

### Class 2 or 5 defective variants

We identified 11 variants belonging to class 2 and/or 5: p.(Asp90Tyr), p.(Glu140Asp), p.(Cys143Trp), p.(Gly149Cys), p.(Gly219del), p.(Cys364Ser), p.(Gln384_Asp386del), p.(Phe403del), p.(Ala431Thr), p.(Val523Met), and p.(Pro608Ser). Of these, p.(Phe403del) exhibited the most severe phenotype, with nearly no detectable LDLR on the cell surface (1 ± 1%). In accordance with the flow cytometry results, the protein with variant p.(Phe403del) appeared solely in the precursor form when analyzed by Western blotting (Fig. 2).

**Fig. 5:**
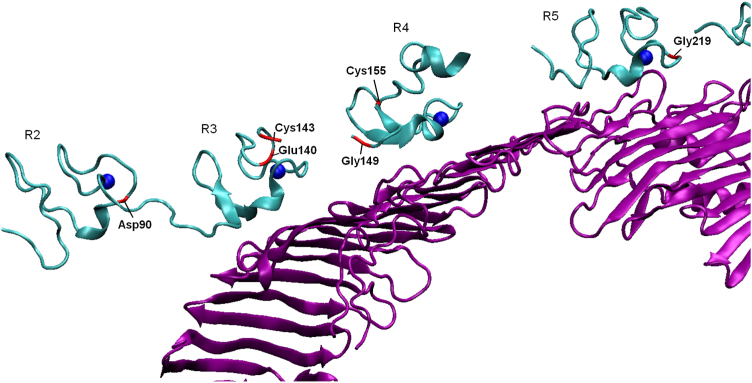
A ribbon diagram of LDLR bound to ApoB, highlighting the studied residues. The model was created in the Visual Molecular Dynamics software (available at http://www.ks.uiuc.edu/Research/vmd/) using the Protein Data Bank structure 9BDT (42, 50). Purple ribbon diagram—ApoB; cyan ribbon diagram—LDLR; and blue spheres—Ca^2+^ ions. LDLR residues mutated in this study are highlighted in red. Repeats of the LDLR-binding domain are labeled R2–R5.

A variant classified as class 2b/5 may also have an additional defect of binding or internalization (class 3 or 4, respectively). To determine whether this is the case, we compared the severity of the LDLR cell surface expression defect and the internalization defect based on the results of our assays. Of the 11 class 2b/5 variants mentioned above, four variants, (p.(Glu140Asp), p.(Cys143Trp), p.(Gly149Cys), and p.(Gly219del), caused a severe defect of LDL internalization despite exhibiting only a slight to moderate defect of LDLR cell surface expression (Fig. 3). The increased defect severity suggests that these variants belong to class 3 or 4 in addition to class 2b/5. All four of these variants were located in the binding domain—p.(Glu140Asp) and p.(Cys143Trp)—in repeat R3, p.(Gly149Cys) in R4, and p.(Gly219del) in R5. Considering that internalization-defective variants are typically found in the NPVY motif in the cytosolic domain, these variants are unlikely to affect internalization. We thus hypothesized that these variants affect LDL binding. To obtain further support for our hypothesis, we mapped the residues of the binding domain mutated in our study (Asp90, Glu140, Cys143, Gly149, Gly219, and the binding-defective control Cys155) on the recently published cryo-EM structure of LDLR bound to ApoB (42). Residues Glu140, Cys143, Gly149, Gly219, and the control Cys155 were located in close proximity to ApoB. In contrast, the p.(Asp90Tyr) variant, which did not exhibit increased severity of the internalization defect compared with the cell surface expression defect in our study, is located in repeat R2, which is not in close contact with ApoB based on this structural model (Fig. 5). Based on these findings, we propose that p.(Glu140Asp), p.(Cys143Trp), p.(Gly149Cys), and p.(Gly219del) belong to class 2b/5 and class 3.

In the case of p.(Val523Met), the results of the Western blot analysis were not entirely consistent with the flow cytometry analysis. The very low mature-to-precursor ratio (0.13 ± 0.04) suggested a more severe defect than was observed by flow cytometry (33 ± 7% LDLR cell surface expression and 67 ± 6% LDL internalization). Previous publications that characterized this variant in patient-derived cells have shown a moderate defect of LDLR activity, which is consistent with our flow cytometry results (17, 35, 36).

### Class 3 or 4 defective variants

This study identified one variant with normal LDLR cell surface expression combined with decreased LDL internalization—p.(Ala540Thr). The result that p.(Ala540Thr) affects LDL binding or internalization may seem surprising considering its location in the EGF precursor homology domain (specifically the beta-propeller), which is a part of neither the binding domain nor the internalization motif. Due to the lack of an assay to specifically study LDL binding, this study could not experimentally distinguish between variants with defective binding and defective internalization; however, we speculated on the variant class based on the variant’s location. Considering the distance of the variant from the internalization motif (NPVY motif) located in the intracellular domain, this variant is unlikely to affect internalization. On the other hand, variants in the beta-propeller have been previously linked to defective binding (51). Moreover, a high-resolution structure of ApoB100 bound to the LDLR extracellular domain showed that the beta-propeller contributes to LDL binding (42). Thus, p.(Ala540Thr) more likely affects LDL binding rather than internalization.

### Variants causing reduced LDLR protein levels

The p.(Leu16Pro) variant differs in location from the other analyzed variants. It affects the signal sequence that is not present in the mature LDLR protein. The signal sequence guides the protein to be synthesized in the ER and is cleaved off afterward (18). This allows the transmembrane domain to be inserted into the ER membrane. Apart from membrane insertion, the unique environment of the ER is also important for disulfide bond formation, N-linked glycosylation, and the incorporation of Ca^2+^ ions into the protein’s structure (reviewed in Ref. (52)). A disruption of the signal sequence could prevent LDLR from reaching the ER, forcing the protein to be synthesized in the cytoplasm, where the LDLR protein cannot achieve correct folding.

As evidenced by this study, the p.(Leu16Pro) variant has a profoundly damaging impact on LDLR function. According to flow cytometry analyses, the amount of LDLR protein on the cell surface was 3 ± 1% of normal, whereas the amount of internalized LDL was 35 ± 4% relative to the benign control. In addition, the protein was nearly undetectable by Western blotting. The failure to detect the protein suggests that this variant either prevents LDLR expression, causes quick degradation of LDLR, or damages LDLR folding in such a way that LDLR becomes undetectable by the antibody.

The p.(Leu16Pro) variant is strikingly similar to another missense variant, p.(Leu15Pro), whose functional study and structural modeling have been previously published (53). Both variants substitute leucine with proline in an alpha-helical region of the signal sequence; this substitution has been shown to disrupt the alpha helix and have a detrimental effect on LDLR function in the case of the p.(Leu15Pro) variant (53). Variant p.(Leu16Pro) is likely to have a similar effect on the structure of the signal sequence as p.(Leu15Pro).

The p.(Cys803Arg) variant is the only transmembrane domain variant analyzed in this study. Variants in the transmembrane domain can reduce LDLR protein levels or prevent the receptor’s membrane incorporation, leading to LDLR secretion outside the cell (11). In our study, variant p.(Cys803Arg) could only be detected as faint bands by Western blotting, indicating that the low cell surface expression (25 ± 5%) as detected by flow cytometry was caused mainly by reduced levels of the LDLR protein rather than LDLR retention in the ER as would be the case for class 2b variants. While ER retention may also occur, the intensity of bands was too low for an accurate analysis of the mature-to-precursor ratio. Reduced LDLR levels could result from decreased protein expression, increased degradation, or misfolding, making the protein unrecognizable by the antibody. Our findings align with a previous publication, which used Western blotting to analyze the expression of variants in the transmembrane domain, including p.(Cys803Arg), and their secretion in the cell culture medium (11). The previous study reported faint bands of both the mature and the precursor form, without detectable secretion of full-length protein in the culture medium. Our study further confirmed the deleterious effect of this variant on LDLR cell surface expression and LDL internalization.

### Classification of variants according to ACMG/AMP guidelines

The clinical significance of genetic variants is most commonly interpreted using a set of guidelines published by the ACMG and AMP (54). An *LDLR*-specific modification of the ACMG/AMP guidelines has been developed by the FH VCEP (25). Based on these guidelines, variants can be divided into five classes: benign, likely benign, pathogenic, likely pathogenic, and VUS. One of the criteria used for variant classification is the results of functional studies. Functional studies are reflected by criteria PS3 (for pathogenicity) or BS3 (for benignity), which can be applied at several levels of strength. The highest strength can be applied if the functional study fulfills the criteria for a level 1 functional study. Chora *et al.* (25) defined a level 1 study as a “Study of the whole LDLR cycle (LDLR expression/biosynthesis, LDL binding, and LDL internalization) performed in heterologous cells (with no endogenous LDLR) transfected with a mutant plasmid.” We believe that the current study can be considered a level 1 functional study encompassing the whole LDLR cycle, even though the study did not include an assay to specifically study LDL binding. Even in the absence of an assay to quantify LDL binding, a binding defect can be detected by the internalization assay because decreased LDL binding necessarily leads to decreased LDL internalization. Altogether, the combination of assays studying LDLR biosynthesis and maturation, LDLR cell surface expression, and LDL internalization allows for the detection of all the defects belonging to the five main classes of *LDLR* variants. We thus applied the strong criteria PS3 and BS3 to the studied variants, except for p.(Ala540Thr) and p.(Asn272Thr), which did not meet thresholds. The p.(Ala540Thr) variant did not qualify for PS3 because the result of the internalization assay was 75% and thus did not fulfill the criterion of a result below 70% according to Chora *et al.* The p.(Asn272Thr) variant had a borderline result of 90% cell surface expression, which prevented it from receiving BS3. However, even with BS3, the variant would currently remain a VUS due to the lack of other evidence. Table 2 shows how the current study contributed to the classification of studied variants based on *LDLR*-specific ACMG/AMP guidelines.

**Table 2:** Contribution of the current functional study to the clinical interpretation of studied *LDLR* variants

Protein change	DNA change	Criterion applied^a^	Variant classification	
			Before current study^b^	After current study^d^
p.(Leu16Pro)	c.47T>C	PS3	**VUS**	**Likely pathogenic**
p.(Asp90Tyr)	c.268G>T	PS3	**Likely pathogenic**	**Pathogenic**
p.(Glu140Asp)	c.420G>C	PS3	Likely pathogenic	**Pathogenic**
p.(Cys143Trp)	c.429C>G	PS3	Likely pathogenic^c^	**Pathogenic**
p.(Gly149Cys)	c.445G>T	PS3	VUS	**Likely pathogenic**
p.(Gly219del)	c.654_656del	PS3	**Pathogenic**	Pathogenic
p.(Asn272Thr)	c.815A>C	None	VUS	VUS
p.(Cys364Ser)	c.1091G>C	PS3	Likely pathogenic	**Pathogenic**
p.(Gln384_Asp386del)	c.1151_1159del	PS3	Likely pathogenic	Likely pathogenic
p.(Phe403del)	c.1207_1209del	PS3	Likely pathogenic	Likely pathogenic
p.(Ala431Thr)	c.1291G>A	PS3	**Pathogenic**	Pathogenic
p.(Val523Met)	c.1567G>A	PS3	Pathogenic	Pathogenic
p.(Ala540Thr)	c.1618G>A	None	**Pathogenic**	Pathogenic
p.(Arg574Leu)	c.1721G>T	BS3	**VUS**	VUS
p.(Pro608Ser)	c.1822C>T	PS3	Likely pathogenic	**Pathogenic**
p.(Cys803Arg)	c.2407T>C	PS3	Likely pathogenic	**Pathogenic**

a Criterion for the interpretation of *LDLR* variants as defined by Chora *et al.* (25), which can be applied based on the results of the current functional study.

b The interpretation of the clinical significance of the *LDLR* variants as reported by the FH VCEP (in bold) or by the majority of submitters in the ClinVar database, as of June 2024.

c Variant p.(Cys143Trp) was not previously reported in ClinVar, so the "before" classification was conducted by the coauthor Dr Lukáš Tichý, who is a member of the FH VCEP.

d Clinical interpretation of studied variants after applying criteria listed in the third column according to *LDLR*-specific ACMG/AMP guidelines (25). Change in classification is highlighted in bold. Change from VUS to another classification is underlined.

### Limitations

Our study had several limitations, one of which was the chosen cell model. We used an LDLR-deficient CHO cell line (CHO-ldlA7) transfected with the human *LDLR* cDNA. However, the effects of *LDLR* variants observed in this cell model might not fully correspond to their effects in human cells. One concern is the interaction between endogenous hamster proteins and human LDLR. As the human LDLR protein (NP_000518.1) is 78% identical to its Chinese hamster counterpart (NP_001233752.1), less efficient processing of the human LDLR by hamster cells may occur.

Another limitation of this cell line is the fact that it has not been derived from hepatocytes. In humans, hepatocytes are responsible for the majority of LDL uptake and are therefore the most physiologically relevant cell type for studying FH (55, 56). Despite this, the CHO-ldlA7 cell line remains the most widely used model for functional studies of the LDLR. Of 45 studies reporting the functional characterization of coding variants in the *LDLR* gene using in vitro methods, 20 studies used CHO-ldlA7 cells and 6 studies used CHO-Trex cells. Hepatic cells were employed by nine studies, all of which used the HepG2 cell line. Other cell lines used included human embryonic kidney 293 cells (nine studies), HeLa cells (three studies), and COS cells (two studies). (The total exceeds 45 because some studies used multiple cell lines.) To ensure that our results would be more easily comparable to previous studies, we decided to use the CHO-ldlA7 cell line.

Another factor affecting the applicability of results was LDLR overexpression. Under physiological conditions, LDLR expression is regulated by intracellular cholesterol levels. In this study, however, LDLR was expressed from a constitutive promoter unresponsive to cholesterol levels. Overexpression might have affected LDLR function and could have contributed to the reduced difference between pathogenic and benign controls in the internalization assay. Moreover, the use of a constitutive promoter prevented the upregulation of defective *LDLR* variants’ expression, which would occur under physiological conditions. However, we decided to maintain equal expression of each variant to allow for an easier comparison of their specific effects on LDLR function.

Another limitation of this study was the absence of an assay specifically designed to assess LDL binding, which would have enabled the experimental differentiation between class 3 and class 4 variants. While a binding defect would manifest as reduced internalization, the distinction between variants class 3 and 4, as reported in Fig. 3, was mainly speculative, based primarily on the location of the variants.

Another shortcoming of the current study was the fact that the cell surface expression assay exhibited a large variability between replicates, particularly between replicates of the WT control. Moreover, WT had the highest mean LDLR cell surface expression of all variants, significantly exceeding that of almost all other variants, including the benign control p.(Thr726Ile). Three of five WT results were higher than the results of any other variant, including one very high value (167%) that could have been an outlier. However, we retained this data point because Dixon’s Q test ruled this value not to be an outlier. Nonetheless, the inclusion of this extreme value could have affected statistical testing. WT plasmids might inherently express more LDLR than mutated plasmids, possibly due to undetected mutations outside the sequenced region that may have arisen during mutagenesis. (The sequenced region included the LDLR-moxGFP fusion gene along with the promoter.) To address this, the p.(Ala391Thr) variant was used as the primary benign control instead of WT.

## Conclusions

Following established methods for the functional characterization of *LDLR* variants (57), we have enhanced the flow cytometry methods for assessing LDLR cell surface expression and LDL internalization by incorporating a monomeric fluorescent tag, which allows for the differentiation of transfected from nontransfected cells. To our knowledge, this is the first functional study of the LDLR that used a fluorescent tag to gate transfected cells during flow cytometry analyses. Several previous studies used LDLR fused to GFP, enhanced GFP, enhanced yellow fluorescent protein, or GFPSpark (26, 27, 31, 53, 58, 59, 60, 61, 62, 63); however, these fluorescent proteins may not be strictly monomeric (64). A nonmonomeric fluorescent tag fused to an ER-localized protein may cause experimental artifacts due to protein oligomerization in the ER, which leads to a change in ER structure (64, 65). We improved the method for flow cytometric functional studies of LDLR by adding a monomeric fluorescent tag optimized for expression in the ER (38) and using its signal to gate transfected cells. The addition of a step to gate transfected cells caused a difference in results for one control variant compared with a previously published study that did not apply such gating, as discussed in the Discussion section addressing the p.(Cys155Tyr) variant.

The methods were optimized using previously characterized benign or pathogenic control variants and subsequently applied to the functional characterization of 16 other *LDLR* variants. Our study presented evidence for a deleterious functional impact of 14 of these variants, whereas two variants exhibited no effect on LDLR function. The results of this study have contributed to the reclassification of two variants from VUS to likely pathogenic and the refinement of the classification of six variants (from likely pathogenic to pathogenic). Two variants remained a VUS even after the functional study due to the lack of other evidence and may be reclassified in the future as more evidence becomes available.

## Data availability

The data that support the findings of this study are available from the corresponding author upon request.

## Conflict of interest

T. F. received honoraria and consultancy fees from Novartis, Sanofi, Sobi, and Medison, outside the submitted work. The other authors declare that they have no conflicts of interest with the contents of this article.

## References

[1] Musunuru K., Hershberger R.E., Day S.M., Klinedinst N.J., Landstrom A.P., Parikh V.N. (2020). Genetic testing for inherited cardiovascular diseases: a scientific statement from the American heart association. Circ. Genom. Precis. Med..

[2] Ference B.A., Ginsberg H.N., Graham I., Ray K.K., Packard C.J., Bruckert E. (2017). Low-density lipoproteins cause atherosclerotic cardiovascular disease. 1. Evidence from genetic, epidemiologic, and clinical studies. A consensus statement from the european atherosclerosis society consensus panel. Eur. Heart J..

[3] Vuorio A., Docherty K.F., Humphries S.E., Kuoppala J., Kovanen P.T. (2013). Statin treatment of children with familial hypercholesterolemia--trying to balance incomplete evidence of long-term safety and clinical accountability: are we approaching a consensus?. Atherosclerosis.

[4] Futema M., Ramaswami U., Tichy L., Bogsrud M.P., Holven K.B., Roeters van Lennep J. (2021). Comparison of the mutation spectrum and association with pre and post treatment lipid measures of children with heterozygous familial hypercholesterolaemia (FH) from eight European countries. Atherosclerosis.

[5] Südhof T.C., Goldstein J.L., Brown M.S., Russell D.W. (1985). The LDL receptor gene: a mosaic of exons shared with different proteins. Science.

[6] Yamamoto T., Davis C.G., Brown M.S., Schneider W.J., Casey M.L., Goldstein J.L. (1984). The human LDL receptor: a cysteine-rich protein with multiple alu sequences in its mRNA. Cell.

[7] Cummings R.D., Kornfeld S., Schneider W.J., Hobgood K.K., Tolleshaug H., Brown M.S. (1983). Biosynthesis of N- and O-linked oligosaccharides of the low density lipoprotein receptor. J. Biol. Chem..

[8] He G., Gupta S., Yi M., Michaely P., Hobbs H.H., Cohen J.C. (2002). ARH is a modular adaptor protein that interacts with the LDL receptor, clathrin, and AP-2. J. Biol. Chem..

[9] Anderson R.G., Goldstein J.L., Brown M.S. (1977). A mutation that impairs the ability of lipoprotein receptors to localise in coated pits on the cell surface of human fibroblasts. Nature.

[10] Maurer M.E., Cooper J.A. (2006). The adaptor protein Dab2 sorts LDL receptors into coated pits independently of AP-2 and ARH. J. Cell Sci..

[11] Strøm T.B., Laerdahl J.K., Leren T.P. (2017). Mutations affecting the transmembrane domain of the LDL receptor: impact of charged residues on the membrane insertion. Hum. Mol. Genet..

[12] Tolleshaug H., Goldstein J.L., Schneider W.J., Brown M.S. (1982). Posttranslational processing of the LDL receptor and its genetic disruption in familial hypercholesterolemia. Cell.

[13] Zou P., Ting A.Y. (2011). Imaging LDL receptor oligomerization during endocytosis using a co-internalization assay. ACS Chem. Biol..

[14] Brown M.S., Anderson R.G., Goldstein J.L. (1983). Recycling receptors: the round-trip itinerary of migrant membrane proteins. Cell.

[15] Basu S.K., Goldstein J.L., Anderson R.G., Brown M.S. (1981). Monensin interrupts the recycling of low density lipoprotein receptors in human fibroblasts. Cell..

[16] Leren T.P. (2014). Sorting an LDL receptor with bound PCSK9 to intracellular degradation. Atherosclerosis.

[17] Hobbs H.H., Brown M.S., Goldstein J.L. (1992). Molecular genetics of the LDL receptor gene in familial hypercholesterolemia. Hum. Mutat..

[18] Hobbs H.H., Russell D.W., Brown M.S., Goldstein J.L. (1990). The LDL receptor locus in familial hypercholesterolemia: mutational analysis of a membrane protein. Annu. Rev. Genet..

[19] Koivisto U.M., Hubbard A.L., Mellman I. (2001). A novel cellular phenotype for familial hypercholesterolemia due to a defect in polarized targeting of LDL receptor. Cell.

[20] Strøm T.B., Tveten K., Laerdahl J.K., Leren T.P. (2014). Mutation G805R in the transmembrane domain of the LDL receptor gene causes familial hypercholesterolemia by inducing ectodomain cleavage of the LDL receptor in the endoplasmic reticulum. FEBS Open Bio..

[21] Strøm T.B., Laerdahl J.K., Leren T.P. (2015). Mutation p.L799R in the LDLR, which affects the transmembrane domain of the LDLR, prevents membrane insertion and causes secretion of the mutant LDLR. Hum. Mol. Genet..

[22] Landrum M.J., Lee J.M., Riley G.R., Jang W., Rubinstein W.S., Church D.M. (2014). ClinVar: public archive of relationships among sequence variation and human phenotype. Nucleic Acids Res..

[23] Tichý L., Freiberger T., Zapletalová P., Soška V., Ravčuková B., Fajkusová L. (2012). The molecular basis of familial hypercholesterolemia in the Czech Republic: spectrum of LDLR mutations and genotype-phenotype correlations. Atherosclerosis.

[24] Tichý L., Fajkusová L., Zapletalová P., Schwarzová L., Vrablík M., Freiberger T. (2017). Molecular genetic background of an autosomal dominant hypercholesterolemia in the Czech Republic. Physiol. Res..

[25] Chora J.R., Iacocca M.A., Tichý L., Wand H., Kurtz C.L., Zimmermann H. (2022). The clinical genome resource (ClinGen) familial hypercholesterolemia variant curation expert panel consensus guidelines for LDLR variant classification. Genet. Med..

[26] Ranheim T., Kulseth M.A., Berge K.E., Leren T.P. (2006). Model system for phenotypic characterization of sequence variations in the LDL receptor gene. Clin. Chem..

[27] Dušková L., Nohelová L., Loja T., Fialová J., Zapletalová P., Réblová K. (2020). Low density lipoprotein receptor variants in the beta-propeller subdomain and their functional impact. Front Genet..

[28] Gudnason V., Patel D., Sun X.M., Humphries S., Soutar A.K., Knight B.L. (1995). Effect of the StuI polymorphism in the LDL receptor gene (Ala 370 to Thr) on lipid levels in healthy individuals. Clin. Genet..

[29] Alves A.C., Azevedo S., Benito-Vicente A., Graça R., Galicia-Garcia U., Barros P. (2021). LDLR variants functional characterization: contribution to variant classification. Atherosclerosis.

[30] Benito-Vicente A., Alves A.C., Etxebarria A., Medeiros A.M., Martin C., Bourbon M. (2015). The importance of an integrated analysis of clinical, molecular, and functional data for the genetic diagnosis of familial hypercholesterolemia. Genet. Med..

[31] Sørensen S., Ranheim T., Bakken K.S., Leren T.P., Kulseth M.A. (2006). Retention of mutant low density lipoprotein receptor in endoplasmic reticulum (ER) leads to ER stress. J. Biol. Chem..

[32] Etxebarria A., Benito-Vicente A., Palacios L., Stef M., Cenarro A., Civeira F. (2015). Functional characterization and classification of frequent low-density lipoprotein receptor variants. Hum. Mutat..

[33] Rubinsztein D.C., Jialal I., Leitersdorf E., Coetzee G.A., van der Westhuyzen D.R. (1993). Identification of two new LDL-receptor mutations causing homozygous familial hypercholesterolemia in a South African of Indian origin. Biochim. Biophys. Acta.

[34] Chang J.H., Pan J.P., Tai D.Y., Huang A.C., Li P.H., Ho H.L. (2003). Identification and characterization of LDL receptor gene mutations in hyperlipidemic Chinese. J. Lipid Res..

[35] Bertolini S., Cassanelli S., Garuti R., Ghisellini M., Simone M.L., Rolleri M. (1999). Analysis of LDL receptor gene mutations in Italian patients with homozygous familial hypercholesterolemia. Arterioscler. Thromb. Vasc. Biol..

[36] Romano M., Di Taranto M.D., Mirabelli P., D'Agostino M.N., Iannuzzi A., Marotta G. (2011). An improved method on stimulated T-lymphocytes to functionally characterize novel and known LDLR mutations. J. Lipid Res..

[37] Sun X.M., Patel D.D., Knight B.L., Soutar A.K. (1997). Comparison of the genetic defect with LDL-receptor activity in cultured cells from patients with a clinical diagnosis of heterozygous familial hypercholesterolemia. The Familial Hypercholesterolaemia Regression Study Group. Arterioscler. Thromb. Vasc. Biol..

[38] Costantini L.M., Baloban M., Markwardt M.L., Rizzo M.A., Guo F., Verkhusha V.V. (2015). A palette of fluorescent proteins optimized for diverse cellular environments. Nat. Commun..

[39] Krieger M., Martin J., Segal M., Kingsley D. (1983). Amphotericin B selection of mutant Chinese hamster cells with defects in the receptor-mediated endocytosis of low density lipoprotein and cholesterol biosynthesis. Proc. Natl. Acad. Sci. U. S. A..

[40] Emmer B.T., Sherman E.J., Lascuna P.J., Graham S.E., Willer C.J., Ginsburg D. (2021). Genome-scale CRISPR screening for modifiers of cellular LDL uptake. PLoS Genet..

[41] Lucero D., Dikilitas O., Mendelson M.M., Aligabi Z., Islam P., Neufeld E.B. (2022). Transgelin: a new gene involved in LDL endocytosis identified by a genome-wide CRISPR-Cas9 screen. J. Lipid Res..

[42] Reimund M., Dearborn A.D., Graziano G., Lei H., Ciancone A.M., Kumar A. (2024). Structure of apolipoprotein B100 bound to the low-density lipoprotein receptor. Nature.

[43] Wurm F.M. (2013). CHO Quasispecies—Implications for manufacturing processes. Processes.

[44] Li C., Zhang J. (2019). Stop-codon read-through arises largely from molecular errors and is generally nonadaptive. PLoS Genet..

[45] van Driel I.R., Goldstein J.L., Südhof T.C., Brown M.S. (1987). First cysteine-rich repeat in ligand-binding domain of low density lipoprotein receptor binds Ca2+ and monoclonal antibodies, but not lipoproteins. J. Biol. Chem..

[46] Jeon H., Blacklow S.C. (2005). Structure and physiologic function of the low-density lipoprotein receptor. Annu. Rev. Biochem..

[47] Pisciotta L., Tarugi P., Borrini C., Bellocchio A., Fresa R., Guerra D. (2010). Pseudoxanthoma elasticum and familial hypercholesterolemia: a deleterious combination of cardiovascular risk factors. Atherosclerosis.

[48] Thormaehlen A.S., Schuberth C., Won H.H., Blattmann P., Joggerst-Thomalla B., Theiss S. (2015). Systematic cell-based phenotyping of missense alleles empowers rare variant association studies: a case for LDLR and myocardial infarction. PLoS Genet..

[49] Junna N., Ruotsalainen S., Ripatti P., FinnGen Ripatti S., Widén E. (2023). Novel Finnish-enriched variants causing severe hypercholesterolemia and their clinical impact on coronary artery disease. Atherosclerosis.

[50] Humphrey W., Dalke A., Schulten K. (1996). VMD: visual molecular dynamics. J. Mol. Graph.

[51] Huang S., Henry L., Ho Y.K., Pownall H.J., Rudenko G. (2010). Mechanism of LDL binding and release probed by structure-based mutagenesis of the LDL receptor. J. Lipid Res..

[52] Gent J., Braakman I. (2004). Low-density lipoprotein receptor structure and folding. Cell Mol. Life Sci..

[53] Pavloušková J., Réblová K., Tichý L., Freiberger T., Fajkusová L. (2016). Functional analysis of the p.(Leu15Pro) and p.(Gly20Arg) sequence changes in the signal sequence of LDL receptor. Atherosclerosis.

[54] Richards S., Aziz N., Bale S., Bick D., Das S., Gastier-Foster J. (2015). Standards and guidelines for the interpretation of sequence variants: a joint consensus recommendation of the American College of Medical Genetics and Genomics and the Association for Molecular Pathology. Genet. Med..

[55] Feingold K.R. (2022). Lipid and lipoprotein metabolism. Endocrinol. Metab. Clin. North Am..

[56] Schmidt H.H., Tietge U.J., Buettner J., Barg-Hock H., Offner G., Schweitzer S. (2008). Liver transplantation in a subject with familial hypercholesterolemia carrying the homozygous p.W577R LDL-receptor gene mutation. Clin. Transplant..

[57] Benito-Vicente A., Uribe K.B., Jebari S., Galicia-Garcia U., Ostolaza H., Martin C. (2018). Validation of LDLr activity as a tool to improve genetic diagnosis of familial hypercholesterolemia: a retrospective on functional characterization of LDLr variants. Int. J. Mol. Sci..

[58] Lin S., Hu T., Wang K., Wang J., Zhu Y., Chen X. (2023). In vitro assessment of the pathogenicity of the LDLR c.2160delC variant in familial hypercholesterolemia. Lipids Health Dis..

[59] Jiang L., Wu W.F., Sun L.Y., Chen P.P., Wang W., Benito-Vicente A. (2016). The use of targeted exome sequencing in genetic diagnosis of young patients with severe hypercholesterolemia. Sci. Rep..

[60] Jiang L., Benito-Vicente A., Tang L., Etxebarria A., Cui W., Uribe K.B. (2017). Analysis of LDLR variants from homozygous FH patients carrying multiple mutations in the LDLR gene. Atherosclerosis.

[61] Wang H., Xu S., Sun L., Pan X., Yang S., Wang L. (2014). Functional characterization of two low-density lipoprotein receptor gene mutations in two Chinese patients with familial hypercholesterolemia. PLoS One.

[62] Rodríguez-Jiménez C., Pernía O., Mostaza J., Rodríguez-Antolín C., de Dios García-Díaz J., Alonso-Cerezo C. (2019). Functional analysis of new variants at the low-density lipoprotein receptor associated with familial hypercholesterolemia. Hum. Mutat..

[63] Rodríguez-Nóvoa S., Rodríguez-Jiménez C., Alonso C., Rodriguez-Laguna L., Gordo G., Martinez-Glez V. (2020). Familial hypercholesterolemia: a single-nucleotide variant (SNV) in mosaic at the low density lipoprotein receptor (LDLR). Atherosclerosis.

[64] Costantini L.M., Fossati M., Francolini M., Snapp E.L. (2012). Assessing the tendency of fluorescent proteins to oligomerize under physiologic conditions. Traffic.

[65] Snapp E.L., Hegde R.S., Francolini M., Lombardo F., Colombo S., Pedrazzini E. (2003). Formation of stacked ER cisternae by low affinity protein interactions. J. Cell Biol..

