# Supplementary material for: Functional characterization of 16 variants found in the LDL receptor gene
Source: J Lipid Res. 2025 Aug 12;66(9):100873. doi: 10.1016/j.jlr.2025.100873 (PMC12454892; doi:10.1016/j.jlr.2025.100873)
Supplement: Supplemental Tables [file mmc1.docx]

# **Supplemental tables**

**Table S1: Primers used for mutagenesis of *LDLR* cDNA inside plasmids.** The table lists primers used to introduce specific variants into *LDLR* cDNA in plasmids pcDNA3-LDLR and pcDNA3-LDLR-moxGFP with the use of QuikChange Lightning mutagenesis kit (Agilent) according to the manufacturer’s instructions. Variant annotation is based on reference sequence NP_000518.1 for protein-level annotation and NC_000019.9(NM_000527.5) for DNA-level annotation.

| **Protein change** | **DNA change** | **Forward primer** | **Reverse primer** |
| --- | --- | --- | --- |
| p.(Leu16Pro) | c.47T>C | cgccttgctccccgccgcggcgg | ccgccgcggcggggagcaaggcg |
| p.(Asp90Tyr) | c.268G>T | cagttctggaggtgctatggccaagtggact | agtccacttggccatagcacctccagaactg |
| p.(Glu140Asp) | c.420G>C | acggctcagacgacgcctcctgcc | ggcaggaggcgtcgtctgagccgt |
| p.(Cys143Trp) | c.429C>G | gaggcctcctggccggtgctcac | gtgagcaccggccaggaggcctc |
| p.(Gly149Cys) | c.445G>T | ggtgctcacctgttgtcccgccagctt | aagctggcgggacaacaggtgagcacc |
| p.(Cys155Tyr) | c.464G>A | cgccagcttccagtacaacagctccacct | aggtggagctgttgtactggaagctggcg |
| p.(Gly219del) | c.654_656del | ggcgctgtgatggccccgactgcaag | cttgcagtcggggccatcacagcgcc |
| p.(Asn272Thr) | c.815A>C | gagcgatgaagttggctgcgttactgtgacactctg | cagagtgtcacagtaacgcagccaacttcatcgctc |
| p.(Cys364Ser) | c.1091G>C | gatcccgacacctccagccagctctgc | gcagagctggctggaggtgtcgggatc |
| p.(Gln384_Asp386del) | c.1151_1159del | tgaggaaggcttcccccacacgaaggcc | ggccttcgtgtgggggaagccttcctca |
| p.(Ala391Thr) | c.1171G>A | cccccacacgaagacctgcaaggctgt | acagccttgcaggtcttcgtgtggggg |
| p.(Phe403del) | c.1207_1209del | cgcctacctcttcaccaaccggcacg | cgtgccggttggtgaagaggtaggcg |
| p.(Arg406Trp) | c.1216C>T | ctcttcttcaccaactggcacgaggtcagga | tcctgacctcgtgccagttggtgaagaagag |
| p.(Arg416Trp) | c.1246C>T | agatgacgctggactggagcgagtacacc | ggtgtactcgctccagtccagcgtcatct |
| p.(Val429Met) | c.1285G>A | ccaacctgaggaacatggtcgctctggac | gtccagagcgaccatgttcctcaggttgg |
| p.(Ala431Thr) | c.1291G>A | tgaggaacgtggtcactctggacacggag | ctccgtgtccagagtgaccacgttcctca |
| p.(Ile441Thr) | c.1322T>C | gtggccagcaatagaacctactggtctgacctg | caggtcagaccagtaggttctattgctggccac |
| p.(Val523Met) | c.1567G>A | aagccaagggccatcatggtggatcctgttc | gaacaggatccaccatgatggcccttggctt |
| p.(Ala540Thr) | c.1618G>A | actgactggggaactcctaccaagatcaagaaag | ctttcttgatcttggtaggagttccccagtcagt |
| p.(Gly565Val) | c.1694G>T | cattcagtggcccaatgtcatcaccctagatctcc | ggagatctagggtgatgacattgggccactgaatg |
| p.(Arg574Leu) | c.1721G>T | tctcctcagtggcctcctctactgggttg | caacccagtagaggaggccactgaggaga |
| p.(Pro608Ser) | c.1822C>T | gaggctggcccactccttctccttggc | gccaaggagaaggagtgggccagcctc |
| p.(Ser691Ter) | c.2072C>A | ccgcagatcaacccccactagcccaagttt | aaacttgggctagtgggggttgatctgcgg |
| p.(Thr726Ile) | c.2177C>T | acccaggagacatccatcgtcaggctaaagg | cctttagcctgacgatggatgtctcctgggt |
| p.(Cys803Arg) | c.2407T>C | tgctcctcgtcttccttcgcctggggg | cccccaggcgaaggaagacgaggagca |
| p.(Asn825Lys) | c.2475C>A | acatcaacagcatcaactttgacaaacccgtctatcaga | tctgatagacgggtttgtcaaagttgatgctgttgatgt |

**Table S2: LDLR cell surface expression analyzed by flow cytometry.** The result of each experiment was converted to percentages by relating the median fluorescence intensity of each sample to that of the benign control p.(Ala391Thr). The table shows the mean of percentages obtained from three or more experiments. A graphical representation of these results can be found in Figure 1A. NA – not applicable. Variant annotation is based on reference sequence NP_000518.1.

| **Variant** | **% LDLR cell surface expression compared to p.(Ala391Thr)** | **Standard deviation** | **Number of biological replicates** |
| --- | --- | --- | --- |
| p.(Leu16Pro) | 3% | 1% | 3 |
| p.(Asp90Tyr) | 23% | 9% | 3 |
| p.(Glu140Asp) | 26% | 3% | 3 |
| p.(Cys143Trp) | 23% | 4% | 3 |
| p.(Gly149Cys) | 48% | 14% | 3 |
| p.(Cys155Tyr) | 26% | 8% | 4 |
| p.(Gly219del) | 45% | 1% | 3 |
| p.(Asn272Thr) | 90% | 5% | 3 |
| p.(Cys364Ser) | 39% | 16% | 3 |
| p.(Gln384_Asp386del) | 52% | 13% | 3 |
| p.(Ala391Thr) | 100% | NA | 22 |
| p.(Phe403del) | 1% | 1% | 3 |
| p.(Arg406Trp) | 46% | 13% | 18 |
| p.(Arg416Trp) | 49% | 6% | 4 |
| p.(Val429Met) | 2% | 1% | 3 |
| p.(Ala431Thr) | 46% | 12% | 3 |
| p.(Ile441Thr) | 3% | 1% | 4 |
| p.(Val523Met) | 33% | 7% | 4 |
| p.(Ala540Thr) | 92% | 5% | 3 |
| p.(Gly565Val) | 1% | 0% | 3 |
| p.(Arg574Leu) | 94% | 11% | 6 |
| p.(Pro608Ser) | 51% | 9% | 4 |
| p.(Ser691Ter) | 1% | 1% | 3 |
| p.(Thr726Ile) | 75% | 13% | 6 |
| p.(Cys803Arg) | 25% | 5% | 3 |
| p.(Asn825Lys) | 79% | 23% | 5 |
| WT | 119% | 31% | 5 |

**Table S3: LDL internalization analyzed by flow cytometry.** The result of each experiment was converted to percentages by relating the median fluorescence intensity of each sample to that of the benign control p.(Ala391Thr). The table shows the mean of percentages obtained from three or more experiments. A graphical representation of these results can be found in Figure 1B. NA – not applicable. Variant annotation is based on reference sequence NP_000518.1.

| **Variant** | **% LDL internalization compared to p.(Ala391Thr)** | **Standard deviation** | **Number of biological replicates** |
| --- | --- | --- | --- |
| p.(Leu16Pro) | 35% | 4% | 3 |
| p.(Asp90Tyr) | 56% | 3% | 3 |
| p.(Glu140Asp) | 31% | 1% | 3 |
| p.(Cys143Trp) | 22% | 8% | 3 |
| p.(Gly149Cys) | 35% | 2% | 3 |
| p.(Cys155Tyr) | 13% | 5% | 3 |
| p.(Gly219del) | 25% | 8% | 3 |
| p.(Asn272Thr) | 108% | 14% | 3 |
| p.(Cys364Ser) | 70% | 5% | 3 |
| p.(Gln384_Asp386del) | 70% | 7% | 3 |
| p.(Ala391Thr) | 100% | NA | 12 |
| p.(Phe403del) | 12% | 3% | 3 |
| p.(Arg406Trp) | 80% | 5% | 10 |
| p.(Arg416Trp) | 75% | 2% | 3 |
| p.(Val429Met) | 25% | 1% | 3 |
| p.(Ala431Thr) | 61% | 4% | 3 |
| p.(Ile441Thr) | 37% | 3% | 3 |
| p.(Val523Met) | 67% | 6% | 3 |
| p.(Ala540Thr) | 75% | 5% | 4 |
| p.(Gly565Val) | 10% | 2% | 3 |
| p.(Arg574Leu) | 98% | 8% | 4 |
| p.(Pro608Ser) | 80% | 3% | 3 |
| p.(Ser691Ter) | 18% | 7% | 3 |
| p.(Thr726Ile) | 99% | 12% | 4 |
| p.(Cys803Arg) | 68% | 3% | 3 |
| p.(Asn825Lys) | 60% | 1% | 3 |
| WT | 103% | 6% | 11 |

**Table S4: Ratio of the mature to the precursor form of LDLR determined by Western blotting.** A graphical representation of these results can be found in Figure 1C. NA – not analyzed. The mature-to-precursor ratio for variants p.(Leu16Pro) and p.(Cys803Arg) was not analyzed because the protein was nearly undetectable. The ratio for variant p.(Ser691Ter) was not analyzed because it was expressed as one band of lowered MW. Variant annotation is based on reference sequence NP_000518.1.

| **Variant** | **Mature-to-precursor ratio (mean of three or more replicates)** | **Standard deviation** | **Number of biological replicates** |
| --- | --- | --- | --- |
| p.(Leu16Pro) | NA | NA | 3 |
| p.(Asp90Tyr) | 1.67 | 0.23 | 3 |
| p.(Glu140Asp) | 1.58 | 0.78 | 4 |
| p.(Cys143Trp) | 0.91 | 0.24 | 3 |
| p.(Gly149Cys) | 1.36 | 0.71 | 4 |
| p.(Cys155Tyr) | 1.11 | 0.17 | 4 |
| p.(Gly219del) | 1.28 | 0.36 | 4 |
| p.(Asn272Thr) | 2.16 | 0.81 | 5 |
| p.(Cys364Ser) | 1.31 | 0.46 | 5 |
| p.(Gln384_Asp386del) | 0.85 | 0.18 | 3 |
| p.(Ala391Thr) | 4.40 | 1.37 | 3 |
| p.(Phe403del) | 0.01 | 0.01 | 4 |
| p.(Arg406Trp) | 0.61 | 0.14 | 8 |
| p.(Arg416Trp) | 0.87 | 0.17 | 3 |
| p.(Val429Met) | 0.03 | 0.02 | 3 |
| p.(Ala431Thr) | 0.57 | 0.23 | 3 |
| p.(Ile441Thr) | 0.04 | 0.01 | 4 |
| p.(Val523Met) | 0.13 | 0.04 | 3 |
| p.(Ala540Thr) | 1.33 | 0.14 | 5 |
| p.(Gly565Val) | 0.01 | 0.02 | 3 |
| p.(Arg574Leu) | 5.56 | 2.80 | 3 |
| p.(Pro608Ser) | 0.59 | 0.17 | 4 |
| p.(Ser691Ter) | NA | NA | 3 |
| p.(Thr726Ile) | 3.54 | 1.15 | 3 |
| p.(Cys803Arg) | NA | NA | 7 |
| p.(Asn825Lys) | 2.65 | 0.29 | 3 |
| WT | 3.30 | 1.25 | 12 |
